# Plant growth-promoting microorganisms as biocontrol agents of plant diseases: Mechanisms, challenges and future perspectives

**DOI:** 10.3389/fpls.2022.923880

**Published:** 2022-10-06

**Authors:** Mohamed T. El-Saadony, Ahmed M. Saad, Soliman M. Soliman, Heba M. Salem, Alshaymaa I. Ahmed, Mohsin Mahmood, Amira M. El-Tahan, Alia A. M. Ebrahim, Taia A. Abd El-Mageed, Shaimaa H. Negm, Samy Selim, Ahmad O. Babalghith, Ahmed S. Elrys, Khaled A. El-Tarabily, Synan F. AbuQamar

**Affiliations:** ^1^Department of Agricultural Microbiology, Faculty of Agriculture, Zagazig University, Zagazig, Egypt; ^2^Department of Biochemistry, Faculty of Agriculture, Zagazig University, Zagazig, Egypt; ^3^Department of Internal Medicine and Infectious Diseases, Faculty of Veterinary Medicine, Cairo University, Giza, Egypt; ^4^Department of Poultry Diseases, Faculty of Veterinary Medicine, Cairo University, Giza, Egypt; ^5^Department of Agricultural Microbiology, Faculty of Agriculture, Beni-Suef University, Beni-Suef, Egypt; ^6^Key Laboratory of Agro-Forestry Environmental Processes and Ecological Regulation of Hainan Province, Hainan University, Haikou, China; ^7^Plant Production Department, Arid Lands Cultivation Research Institute, The City of Scientific Research and Technological Applications, Alexandria, Egypt; ^8^Jiangsu Key Laboratory for Microbes and Genomics, School, of Life Sciences, Nanjing Normal University, Nanjing, China; ^9^Department of Soils and Water, Faculty of Agriculture, Fayoum University, Fayoum, Egypt; ^10^Department of Home Economic, Specific Education Faculty, Port Said University, Port Said, Egypt; ^11^Department of Clinical Laboratory Sciences, College of Applied Medical Sciences, Jouf University, Sakaka, Saudi Arabia; ^12^Medical Genetics Department, College of Medicine, Umm Al-Qura University, Makkah, Saudi Arabia; ^13^Soil Science Department, Faculty of Agriculture, Zagazig University, Zagazig, Egypt; ^14^Department of Biology, College of Science, United Arab Emirates University, Al-Ain, United Arab Emirates; ^15^Khalifa Center for Genetic Engineering and Biotechnology, United Arab Emirates University, Al-Ain, United Arab Emirates; ^16^Harry Butler Institute, Murdoch University, Murdoch, WA, Australia

**Keywords:** biofertiIizers, biopestcide, crop yield, disease suppression, pathogen suppression, plant growth-promoting rhizhobacteria

## Abstract

Plant diseases and pests are risk factors that threaten global food security. Excessive chemical pesticide applications are commonly used to reduce the effects of plant diseases caused by bacterial and fungal pathogens. A major concern, as we strive toward more sustainable agriculture, is to increase crop yields for the increasing population. Microbial biological control agents (MBCAs) have proved their efficacy to be a green strategy to manage plant diseases, stimulate plant growth and performance, and increase yield. Besides their role in growth enhancement, plant growth-promoting rhizobacteria/fungi (PGPR/PGPF) could suppress plant diseases by producing inhibitory chemicals and inducing immune responses in plants against phytopathogens. As biofertilizers and biopesticides, PGPR and PGPF are considered as feasible, attractive economic approach for sustainable agriculture; thus, resulting in a “win-win” situation. Several PGPR and PGPF strains have been identified as effective BCAs under environmentally controlled conditions. In general, any MBCA must overcome certain challenges before it can be registered or widely utilized to control diseases/pests. Successful MBCAs offer a practical solution to improve greenhouse crop performance with reduced fertilizer inputs and chemical pesticide applications. This current review aims to fill the gap in the current knowledge of plant growth-promoting microorganisms (PGPM), provide attention about the scientific basis for policy development, and recommend further research related to the applications of PGPM used for commercial purposes.

## Introduction

Plant pathogens and pests can have a large impact on agricultural productivity. Plant diseases reduce yields by 21–30% in several important crops worldwide (Savary et al., 2019). Meanwhile, certain plant pathogens have developed long-term resistance against chemical management (Lucas, 2011). Some economically important plant diseases have become more prevalent. Dependence on chemical pesticides has become one of the most pressing challenges to global environmental sustainability and public health (Fones et al., 2020). Because many of insecticides are difficult to break down into simpler components that are less dangerous, toxic residues remain in the soil; thus, posing health concerns (Gilden et al., 2010). Awareness of the environmental and health risks associated with synthetic chemical pesticides is highly recommended for sustainable crop management and less used chemicals (Donley, 2019).

Synthetic agrochemicals have been considered unsustainable, causing the quest for more environmentally friendly alternatives. The focus of modern agriculture research has turned to farm practices. Plant growth-promoting rhizobacteria (PGPR) are effective, environmentally safe, and non-toxic naturally occurring microorganisms than can serve as a promising alternative to chemical pesticides. Besides, environmental factors can affect agricultural productivity; thus, this may worsen the scenario in a variety of ways. We have many reasons to take serious actions toward plant disease control management to improve our health and reduce the effects of environmental stresses (Chaloner et al., 2021). Biological control provides one of the most economical and long-term effective strategies for managing plant diseases and reducing crop loss.

Recent advances in our understanding to plant growth-promoting microorganisms (PGPMs) warrant a proper scientific evaluation of the relationship between the properties of PGPMs and their impact on plant growth, yield, and resistance/tolerance to biotic and abiotic stresses. In addition, this review study builds on a growing body of literature concerning some potential implementations of PGPMs in sustainable agriculture. Here, the aim is to provide a state-of-knowledge review reporting the effects of PGPMs on plants and finding solutions to the challenges that face microbial biological control agents (MBCAs) when applied on a large scale compared with those of chemicals.

## PGPR as promising biocontrol agents

Soil is a complex ecosystem containing various groups of microorganisms, including bacteria, fungi, protists, and animals (Müller et al., 2016). These microorganisms play key roles in plant development, nutrient regulation, and biocontrol activities. They settle in the rhizosphere and endo-rhizosphere of plants, where they use a variety of direct and indirect processes to support plant growth. Lyu et al. (2020) have stated that the phytomicrobiome (plant-associated microorganisms) can provide competitive, exploitative, or neutral alliances with plants; thus, affecting crop yield. Recently, scientists have looked deeply into employing beneficial PGPR to inhibit phytopathogens and promote plant growth (Qiao et al., 2017; Alwahshi et al., 2022). A key part of this might be attributed to the enhancement of target specificity between PGPR and the plant species (Lommen et al., 2019).

According to Zgadzaj et al. (2016), rhizosphere microbiome refers to bacterial, archaeal and fungal communities as well as their genetic material closely surrounding plant root systems. Microorganisms can indirectly impact crop health and phenotypic plasticity by influencing the growth of plants and defense responses due to their co-evolution with plants on a large scale (Goh et al., 2013). The rhizosphere is home to various microorganisms that provide steady PGPR supplies (Antoun and Kloepper, 2001). The phytomicrobiome includes the bacterial population that colonize the rhizosphere, on the root surface, and between the root cortex cells (Inui Kishi et al., 2017). Since plants can first colonize the terrestrial environments, PGPR have co-evolved with related plants; resulting in synergistic host plants’ relationships (Gouda et al., 2018). The effects, methods, and possibility for successfully applying PGPR to agricultural plant production in controlled situations have been the subject of numerous studies. This is critical for developing more widely used methods of biological control that consider field settings.

Safety and quality control are more crucial in vegetable cultivation since we use them less processed or unprocessed, and they have impact on health. PGPR are more achievable under greenhouse conditions. Because of the controlled environment, a significant number of prospective BCA has been discovered, and maybe ready for placement (Singh et al., 2017); thus, they have been confirmed to be successful in greenhouse investigations (Liu et al., 2018). *Bacillus subtilis, Bacillus amyloliquefaciens* and *Pseudomonas stutzeri* are among those shown to achieve success in root colonization as well as prevention of the pathogen *Phytophthora capsici* in cucumber (Islam et al., 2016). At the post-harvest stage, *B. subtilis* can protect tomato fruits from infection by *Penicillium* sp. and *Rhizopus stolonifer* (Punja et al., 2016).

Under greenhouse conditions, *B. amyloliquefaciens* isolates, diminish *Fusarium oxysporum* causing *Fusarium* wilt disease (Gowtham et al., 2016). In controlled situations, PGPR -as BCAs- are effective, indicating their role in greenhouse production systems and their efficacy in commercial horticulture. It is not necessary to distinguish the indirect PGPR pathways for pathogen infection avoidance and plant growth promotion under abiotic stresses. In addition, PGPR with biocontrol activities that also enhance plant growth would be more effective in practice. Plant tolerance to abiotic conditions and resistance to phytopathogens causing plant diseases can be improved by PGPR (Bhat et al., 2020; Leontidou et al., 2020). Some strains benefit plants’ coping with stress and flourishing in abiotic environments (Goswami and Deka, 2020).

While most researchers have reported PGPR under these controlled conditions, few of them have investigated their effectiveness as BCAs, especially when combined with an abiotic stress. This is a critical factor in field biocontrol, and when climate change affects the ecosystem. The long synergism between PGPR and plant may deliver various benefits to the host plant (Fan et al., 2020).

## Biocontrol mechanisms using PGPR

PGPR can enhance the availability of certain nutrients [phosphate solubilization and nitrogen (N_2_) fixation], or synthesize the phytohormones [indole-3 acetic acid (IAA), ethylene (ET), jasmonic acid (JA), gibberellic acid (GA), and cytokinins (CKs); Mengiste et al., 2010; Vejan et al., 2016; Gouda et al., 2018; Sham et al., 2019].

**Figure 1:**
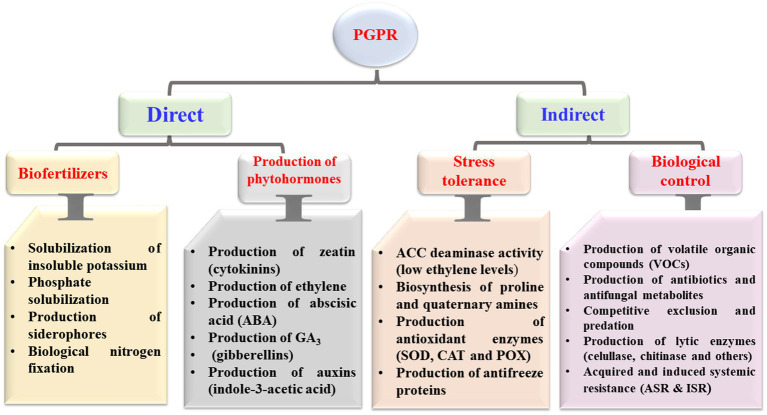
Direct and indirect mechanisms mediated by plant growth-promoting rhizobacteria (PGPR) with beneficial effects on host plants. ACC, 1-aminocyclopropane-1-carboxylic acid; SOD, superoxide dismutase; CAT, catalase; and POX, peroxidase.

PGPR colonizing a host plant can stimulate its growth through direct and indirect mechanisms (Figure 1). Direct mechanisms include the production of plant hormones, solubilization of phosphates, and increased uptake of iron. Indirect effects include antibiotics production, nutritional competition, parasitism, pathogen toxin inhibition, and induced resistance (Elnahal et al., 2022). The attitude of “PGPR” in creating phytohormones, molecules of signaling metabolites, and related substances describe how plants protect themselves from drought as an example of abiotic stress and salinity (Jochum et al., 2019). According to Abbas et al. (2019), PGPR may also alter the shape of the roots, resulting in increased root surface and improved root performance. In addition, PGPR can compete with other bacteria by colonizing rapidly and accumulating a greater supply of nutrients, preventing other organisms from growing (Salomon et al., 2017, Abd El-Mageed et al., 2020). PGPR have different strategies to colonize, of which each is tied to a particular host (Choudhary et al., 2011). In general, pathogen infections can be suppressed by using antibiotics and antifungal metabolites; thus considered a well-known direct biological control strategy (Raaijmakers et al., 2002). Bacteriocins, antibacterial proteins, and enzymes are examples of antimicrobial peptides (Compant et al., 2005). Antibiotics are small antimicrobial molecules produced by PGPR that can inhibit the process of metabolic or growth activities of microbial pathogens (Duffy et al., 2003). These antibiotics, which are mostly strain-specific, can target the ribosomal RNA (rRNA), alter the membrane structure, and damage the cell walls of bacterial pathogens (Abriouel et al., 2011; Maksimov et al., 2011; Nazari and Smith, 2020).

Many bacteria produce bacteriocins, where some have a greater variety of inhibitory activities than others (Abriouel et al., 2011). Siderophores are specialized chelating agents of ferric iron that inhibit phytopathogens from gaining access to iron; thus, maintaining plant health particularly in iron-deficient environments (Shen et al., 2013). PGPR can manage various plant diseases by depriving pathogens of iron, thereby reducing disease development and generating extracellular siderophores (Radzki et al., 2013). Bacteriocins, siderophores, and antibiotics have thus been identified as the three supreme operative approaches for potential biocontrol prior to *in vivo* applications (Kloepper et al., 1980). Several studies have investigated PGPR as a potential plant disease management tool to synthesize plant-beneficial metabolites such as siderophores (Subramanian and Smith, 2015).

PGPR can indirectly increase crop stress tolerance. Signal chemicals, such as phytohormones and specialized signal molecules, enable plant-to-microbe and microbe-to-plant communication (Lyu et al., 2020). The control and regulation of activities in the holobiont include the host plant and the “specific” phytomicrobiome (the plant-phytomicrobiome interaction). Two microbe-to-plant signals, lipo-chitooligosaccharides (LCOs) and thuricin17 (TH17), enhance stress tolerance in different plant species (Lyu et al., 2020). Resistance-inducing and antagonizing PGPR might be useful as new inoculants with combinations of different mechanisms of action, leading to a more efficient use for biocontrol strategies and plant growth promotion (Glick, 1995).

PGPR can also produce volatile organic compounds (VOCs) that play a significant role in plant growth and induced systemic resistance (ISR) to pathogens (Raza et al., 2016). Beneduzi et al. (2012) found that PGPR can trigger ISR as a strategy to improve disease resistance of plants. Roots colonization by arbuscular mycorrhizal fungi (AMF) and certain strains of non-pathogenic bacteria can improve plant resistance to biotic stresses (Mauch-Mani et al., 2017; Pérez-de-Luque et al., 2017). ISR triggered by PGPR and plant growth-promoting fungi (PGPF) can be found in a wide range of plant taxa (Bhattacharyya and Jha, 2012). Systemic acquired resistance (SAR); however, can be activated by a pathogen infection (Gao et al., 2015). Salicylic acid (SA) signaling is associated with the production of pathogenesis-related (PR) genes. Unlike SAR, ISR functions independently of SA, but requires responses to ET and JA. This can be achieved by the induction of defense-related gene expression; although is not always associated with induced PR proteins (Mathys et al., 2012).

Previous studies have identified ISR to stimulate PGPR via the SA-dependent pathway rather than the JA/ET-dependent pathway (Takishita et al., 2018). Other plant hormones, such as auxins, GA, CKs and brassinosteroids, may also contribute to plant immunity (Nakashita et al., 2003; Kazan and Manners, 2009; Giron et al., 2013; Rady et al., 2021). Hormonal crosstalk is thought to allow the cultivation and exert their immunological growth and defense reactions (Pieterse et al., 2014; AbuQamar et al., 2017; Sham et al., 2017).

PGPR are involved in diverse mechanisms to enhance plant growth and/or act as BCAs. Crop production promotion and disease management could be investigated together to ensure sustainability and cost-effectiveness of agricultural systems. Thus, effective PGPR strains can promote stress tolerance and nutrient absorption, plant development, and battling fungal/bacterial diseases. Thus, this appears to be a win-win situation to the PGPR strain and the host plant.

## Challenges of employing PGPR as BCAs

PGPR-based biocontrol provides effective and long-lasting disease management. Europe and the United States are the most promising marketplaces for biocontrol products, followed by South America (Barratt et al., 2018). Although many PGPR have been tested *in vitro* and commercially proven as BCAs, new biocontrol products have been released from research activities carried out in the United States and Europe (Glick, 2012; O’Brien, 2017; Rosier et al., 2018). In general, the market of BCAs and their products is growing; yet, it is not well-adopted compared with chemical pesticides as the most common crop management method (Mishra et al., 2015).

Before being publically accepted/registered as a commercial BCA, there are certain requirements/needs that have to be taken into consideration (Bashan et al., 2014). As such, researchers should improve the efficacy of BCAs to manage certain disease(s). This can be achieved by having a BCA that has as many beneficial characteristics and mechanisms of action as possible. Such characteristics may include, but not limited to, the ability of the BCA to grow fast *in vitro*, produce a wide range of bioactive metabolites, possess high rhizosphere competence abilities, enhance plant growth performance, be environmentally safe, have the compatibility with other rhizobacteria/fungi, and be tolerant to abiotic stresses (Lyu et al., 2020). Successful colonization of root tissues and/or the rhizosphere is a critical component for any PGPR strain to be an effective BCA; thus, to perform well against plant pathogens. On the other hand, performance of the inoculated PGPR may vary, depending on the survival rate in the soil, crop compatibility, interaction with other local microbial species and the environmental factors (Vejan et al., 2016). Survival and colonization are major components when identifying effective BCA isolates. *In vitro* antagonism experiments are often used to investigate the effect of bacterial isolates on certain diseases, prior to greenhouse and/or field trials (Bashan et al., 2014).

Performance of PGPR is generally assessed according to the geographical areas, soil types, host crop species, and under various environmental conditions (Choudhary et al., 2011). BCA growth is often easier to monitor under controlled conditions, i.e., greenhouses. The preference of this stage by most researchers could be attributed to the stability of environmental conditions. Greenhouse experiments evaluating the performance of BCAs under controlled conditions can provide strong theoretical and practical support for the application of PGPR in the field. Thus, this ensures the feasibility and efficacy of PGPR for commercial horticulture production, disease management and climate change conditions such as those found under field conditions.

PGPR stability is also influenced by the method, formulations, transportation, and storage conditions. To achieve high levels of the BCA survival (McIntyre and Press, 1991), one should improve the formulation technology (Lobo et al., 2019), increase the shelf-life of the BCA product (Carrasco-Espinosa et al., 2015), optimize the production of targeted microbial types (Zhang et al., 2019) and achieve low-cost production at large scales (Kang et al., 2017). Many scientists have attempted to extend the shelf-life of PGPR by decreasing the storage temperature and/or modifying the combinations of additives (Lee et al., 2016; Berger et al., 2018). Extensive research on the risks and benefits of BCAs is also required, because agricultural disease management approaches rely on this balance.

Due to the diverse modes of action, identification, characterization, the registration of promising PGPR strains take time and require academic-industry collaborations. Using natural sources (e.g., BCAs) to control pathogens also poses a set of legal and ethical issues that may threaten the local biodiversity (Hajek et al., 2016). In that regard, new species/populations of BCAs have been restricted from entering specific countries. For commercial uses, the application of PGPR in protected environments such as greenhouses is much easier, due to a more isolated and controlled environment delivery and potentially less negative ecological consequences. Another challenge that is linked to the widespread implementation of PGPR-based biocontrol is the regulatory problems. Currently, each country has its regulatory system that greatly vary among them (Bashan et al., 2014).

High development costs for new commercial BCAs, for example, have been identified as a barrier to the BCA industry’s expansion in Australia (Begum et al., 2017). The high regulatory expenses of importing new BCAs into Australia is one of the most serious challenges. BCA registration requies tight coordination among governmental institutes, universities, and industrial sectors to facilitate the assessment and commercialization of new BCAs and their products. The shortage of programs for financial and ecological benefits can also be added as a challenging problem (Heimpel et al., 2013). For global marketing and local practical applications, commercialization should follow international legislation. The International Biological Control Organization (IOBC) have gathered academicians, researchers and practitioners from different sectors/fields to identify the barriers and provide recommendations to overcome these limitation (Barratt et al., 2018).

When compared to chemical pesticides, which are more reliable and predictable, farmers could notice little or no economic gain. Such programs, including local seminars, training workshops and free conferences may increase awareness about the application of BCA in specific farming areas. Finally, PGPR-based biocontrol can hold a lot of promises to reduce agrochemicals use in agriculture. The widespread use of PGPR as BCAs requires massive effort from regulatory bodies and crop growers to convince the public and earn their trust in the capacity of the new BCA products to manage diseases and increase crop yields. High-value crop production in greenhouses could be an ideal place to test the efficacy of PGPR as BCAs in response to different abiotic stresses. Based on recent successful greenhouse trials, BCAs can be used in the field for managing disease and associated agricultural plant growth enhancement (Alwahshi et al., 2022).

## Rhizobacteria as BCAs

In the past few decades, rhizobacteria have gained attention when applied to grains, seeds, roots, and/or soils to help the plant grow and develop. Rhizobacteria are important for N_2_ fixation, promotion of plant growth, and biological control of plant pathogenic microorganisms. Recently, various microbial species are presently used in bacterization, containing *Azospirillum*, *Azotobacter*, *Bacillus*, *Rhizobium*, *Serratia*, *Stenotrophomonas*, *Streptomyces Acinetobacter*, *Agrobacterium*, *Alcaligenes*, *Arthrobacter*, *Bradyrhizobium*, *Frankia*, *Pantoea*, *Pseudomonas*, and *Thiobacillus* (Whipps, 2001). Many plant diseases associated with nematodial, bacterial and fungal infections have been reported to be managed by PGPR. The use of BCAs has been controversial in suppressing nematode populations because other soil microorganisms and the host plant can be adversely affected. To manage diseases associated with plant-parasitic nematodes a combination of biological management, nematicides, organic soil amendments, and crop rotation have been used (Timper, 2011). *In vitro* culture filtrates of a strain of *Pseudomonas* sp. can suppress juvenile mortality of *Meloidogyne javanica*; thus, considerably reduce root gall and nematode population, and enhance plant development and yield (Nasima et al., 2002). Furthermore, inoculations with *Bacillus* spp. affect nematode behavior and feeding (Viaene et al., 2006). *Pseudomonas striata*, *Pseudomonas fluorescens*, and *B. subtilis* strains also overturn the population of nematodes (Table 1; Khan et al., 2012). Root-knot nematode (RKN) infestations have been successfully managed via biological management using *Bacillus* isolates (Lee and Kim, 2016). Few studies have reported the endophytic *P. fluorescens* and *Bacillus* spp. to promote systemic resistance in crops against nematodes owing to the increased activities of phenylalanine ammonia-lyase, polyphenol oxidase, and peroxidase, as defense-related enzymes for producing antagonistic chemicals and altering explicit root exudates such as amino acids and polysaccharides (Abbasi et al., 2014). *P. fluorescens* isolates increased defense enzymes in tomatoes resistant to RKN (Kavitha et al., 2013). In comparison with the control, the application of *P. fluorescens* and *Paecilomyces lilacinus* resulted in low nematode community in roots, tubers and soils (Mohan et al., 2017).

**Table 1:** Bacterial and fungal plant growth-promoting strains used as biocontrol agents against plant pathogenic microorganisms.

Host	Pathogen	Disease	PGP strains Bacteria/Fungi	References
**I. Bacteria**				
Soybean	*Fusarium solani, Macrophomina phaseolina*	Root rot	*Bradyrhizobium* sp.	Parveen et al., 2019
	*Sclerotinia sclerotiorum*	White mold	*Butia archeri*	Vitorino et al., 2020
Pigeon pea	*Fusarium udum*	*Fusarium* wilt	Rhizobacteria spp.	Dukarea and Paulb, 2021
	*Erwinia tracheiphila*		*Glutamicibacter* spp. FBE-19	Fu et al., 2021
Apple	*Mucor piriformis*	*Mucor* rot	*Pseudomonas fluorescens*	Wallace et al., 2018
Rice	*Meloidogyne incognita*	Root-knot nematode	*Trichoderma citrinoviride* Snef1910	Tariq et al., 2020
	*Magnaporthe oryzae*	Blast disease	*Pseudomonas putida* BP25	Ashajyothia et al., 2020
	*Xanthomonas axonopodis pv. glycines*	Bacterial pustule	*Pseudomonas parafulva* JBCS1880	Kakembo and Lee, 2019
	*Phytophthora capsici*	NA	*Pseudomonas*, *Burkholderia*	Khatun et al., 2018
	*Xanthomonas oryza*	Bacterial leaf blight	*Bacillus subtilis strain* GBO3	Faizal Azizi and Lau, 2022
Strawberry	*Macrophomina phaseolina*	Charcoal rot disease	*Azospirillum brasilense*	Viejobueno et al., 2021
	*Botrytis cinerea*	Gray mold	*Bacillus amyloliquefaciens* Y1	Maung et al., 2021
Cotton	*Macrophomina phaseolina*	Charcoal rot disease	*Pseudomonas aeruginosa* and *Sargassum ilicifolium*	Rahman et al., 2017
	*Colletotrichum gossypii*	Ramulosis disease	*Bacillus amyloliquefaciens,* and *Bacillus velezensis*	Ferro et al., 2020
Citrus fruit	*Penicillium digitatum*	Blue mold	*Bacillus megaterium*	Mohammadi et al., 2017
Oil seed rape	*Sclerotinia sclerotiorum*	*Sclerotinia* stem rot	*Trichoderma atroviride*	Hidayah et al., 2022
*Brassica campestris* L.		Sclerotiniose	*Bacillus thuringiensis*	Wang et al., 2020
Canola		Sclerotinia stem rot	*Paenibacillus chlororaphis*	Savchuk and Fernando, 2004
Maize	*Fusarium graminearum*	Stalk rot	*Bacillus methylotrophicus*	Cheng et al., 2019
Wheat	*Stagonospora nodorum*	*Stagonospora nodorum* blotch	*Bacillus subtilis* 26DCryChS	Maksimov et al., 2020
	*Rhizoctonia solani* AG-8	Wheat root pathogen	*Bacillus subtilis*	Zhang et al., 2021a
Pepper	*Phytophthora capsici*	Blight and fruit rot	*Bacillus licheniformis* BL06	Li et al., 2020
Tomato	*Fusarium oxysporum* f. sp. *lycopersici*	*Fusarium* wilt	*Brevibacillus brevis*	Liu et al., 2022
	*Rhizoctonia solani*	Damping-off	*Burkholderia cepacia* BY	Al-Hussini et al., 2019
Mango	*Lasiodiplodia theobromae*	Dieback	*Streptomyces samsunensis* UAE1, *Streptomyces cavourensis* UAE1, *Micromonospora tulbaghiae* UAE1	Kamil et al., 2018
Tea	*Colletotrichum* sp,	Shoot necrosis	*Trichoderma camelliae*	Chakruno et al., 2022
Date palm	*Fusarium solani*	Sudden death syndrome	*Streptomyces polychromogenes* UAE2, *Streptomyces coeruleoprunus* UAE1	Alblooshi et al., 2022
			*Streptomyces tendae* UAE1, *Streptomyces violaceoruber* UAE1	Alwahshi et al., 2022
	*Thielaviopsis punctulata*	Black scorch	*Streptomyces globosus* UAE1	Saeed et al., 2017
Royal poinciana	*Neoscytalidium dimidiatum*	Stem canker	*Streptomyces rochei* UAE2, *Streptomyces coelicoflavus* UAE1 and *Streptomyces antibioticus* UAE1	Al Raish et al., 2021
			*Streptomyces griseorubens* UAE2	Al Hamad et al., 2021
Banana	*Fusarium* spp.	Postharvest diseases	*Trichoderma* spp.	Snehalatharani et al., 2021
**II. Fungi**				
Rice	*Helminthosporium oryzae, Bipolaris oryzae*	Leaf brown spot	*Trichoderma viride, Trichoderma harzianum, Trichoderma hamatum*	Khalili et al., 2012; Mau et al., 2022
Scorzonera	*Alternaria scorzonerae, Fusarium culmorum*	Root and stem rot	*Trichoderma harzianum T-22*	Patkowska, 2021
			*Trichoderma* spp.	Bilesky-José et al., 2021
	*Sclerotinia sclerotiorum, Botrytis cinerea, Fusarium solani, Fusarium cucurbitae, Pythium aphanidermatum, Rhizoctonia solani, Mycosphaerella melonis*		*Trichoderma aggressivum*	Sánchez-Montesinos et al., 2021
Tobacco	*Fusarium, Rubrobacter,* and *Talaromyces* spp.	Root rot	*Paenibacillus polymyxa Trichoderma harzianum*	Yao et al., 2021
Okra	*Meloidogyne incognita*	Root-knot disease	*Trichoderma virens*	Tariq et al., 2018
Beans	*Botrytis cinerea*	Chocolate spot	*Trichoderma atroviride*	Yones and kayim, 2021
	*Sclerotinia sclerotiorum*	Wild mold	*Trichoderma asperellum*	Zapata-Sarmiento et al., 2020
Onion	*Sclerotium cepivorum*	White rot		Rivera-Méndeza et al., 2020
Tomato	*Colletotrichum gloeosporiodes*	Crop loss	*Trichoderma longibranchiatum*	De la Cruz-Quiroz et al., 2018
Cabbage	*Fusarium oxysporum*	Cabbage *Fusarium* wilt	*Rhizobactrin*	Khafagi et al., 2020
	*Sclerotium sclerotiorum*	Cabbage wilt	*Trichoderma hamatum*	Jones et al., 2014
Cocoa	*Phytophthora Palmivora*	Black pod	*Aspergillus fumigates*	Adebola and Amadi, 2010
Tomato	*Fusarium oxysporum f.* sp. *lycopersici*	Wilt	*Penicillium oxalicum*	Murugan et al., 2020
	*Rhizophagus intraradices*	*Verticillium* wilt	*Penicillium pinophilum*	Ibiang et al., 2021
	*Meloidogyne javanica*	Root-knot disease	*Paecilomyces lilacinus*	Hanawi, 2016
*Vigna radiata*	*Meloidogyne incognita*		*Purpureocillium lilacinum*	Khan et al., 2019
Pineapple	*Meloidogyne javanica*		*Purpureocillium lilacinum*	Kiriga et al., 2018
Carrot			*Pochonia chlamydosporia*	Bontempo et al., 2017
Kiwi	Postharvest diseases	Kiwi fruit wound	*Debaryomyces hansenii*	Sui et al., 2021
	Soil-borne pathogens		Rhizosphere	Tsegaye et al., 2018
Tomato	*Sclerotium rolfsii*	Southern blight	*Stenotrophomonas maltophilia* PPB3	Sultana and Hossain, 2022
	*Phytophthora infestans*	Late blight	*Rhizopus* spp.	Agbor et al., 2021
Peaches	*Monilinia laxa*	Postharvest fruit decay	*Aureobasidium pullulans*	Di Francesco and Baraldi, 2021
Sweet potato	*Ceratocystis fimbriata*	Black rot disease	*Pseudomonas chlororaphis* subsp. *aureofaciens* SPS-41	Zhang et al., 2021b
Rice	*Pyricularia oryzae*	Rice blast fungus	Rhizobacteria	Nabila and Kasiamdari, 2021
Chickpea	*Rhizoctonia bataticola*	Chickpea dry root rot	*Bacillus subtillis*	Chiranjeevi et al., 2021

According to Jha et al. (2015), losses resulting from post-fresh fruit and vegetable harvest in India ranged between 4.6% and 15.9%. Although fungicides can inhibit the growth of phytopathogens, their use causes problems to the environment as well as the human and animal health (Nunes, 2012). The most environmentally acceptable practice to control post-harvest fungal diseases is by using BCAs. In general, BCAs can protect plants from fungal diseases, and are currently a viable option to manage post-harvest diseases associated with plant pathogens (Ghazanfar et al., 2016). In agriculture, BCAs can offer a number of advantages, including the reduction in the causing agents, farming preservation, minimum labor, soil, water plant contamination, and waste management difficulties (Torres et al., 2016). Fungal species, such as *Alternaria*, *Aspergillus Penicillium*, and *Fusarium* producing mycotoxins, are harmful to green vegetables and cause post-harvest diseases. Mycotoxins, such as fumonisin, ochratoxins, aflatoxins and other toxins, are released in vegetables and fruits infected with the fungal pathogens, *Fusarium*, *Alternaria*, and *Aspergillus* (Sanzani et al., 2016). The use of biopriming and pelletizing techniques of *Serratia plymuthica* HRO-C48 alongside *Verticillium dahliae* in canola plants revealed a significant biocontrol (Muller and Berg, 2008); thus, providing evidence of the ability of BCAs to manage diseases comparable to chemical fungicides.

*Bacillus* spp. produce a variety of compounds involved in the biocontrol of phytopathogens on various plants, including potato, rice, tomato, wheat, groundnut, brinjal, chickpea, and cucumber (Peng et al., 2014). *Bacillus* sp. BS061 isolate can mitigate the effect of *Botrytis cinerea* to reduce the occurrence of gray mold and powdery mildew diseases in strawberry and cucumber (Kim et al., 2013). Park et al. (2013) found that *Pectobacterium carotovorum* SCC1 can manage soft rot disease in tobacco plants when conjugated with *B. subtilis* strain B4 and BTH fungicides. The root-knot and root rot pathogens are often suppressed when *Pseudomonas* spp. are used as BCAs (Habiba et al., 2016). Farhat et al. (2017) revealed that PGPR isolates had antifungal activities in mungbean plants against *Rhizoctonia solani*, *Macrophomina phaseolina*, *F. solani*, and *F. oxysporum*. These isolates can also be used to prevent fungal infections that cause root rot disease. The application of the bacterial BCA, *Pseudomonas aeruginosa*, can manage anthracnose in the chili pepper against the causal pathogen *Colletotrichum capsici* (Jisha et al., 2018). *P. aeruginosa* can also induce systemic resistance of chili pepper to anthracnose.

Synthetic chemical pesticides are mainly used for post-harvest disease management. Thus, this may lead to plant pathogen resistance, soil deterioration, and toxicological hazards for the humans and the environment. Nowadays, a general trend, as a result, has been shifted toward finding an alternative to the use of agrochemicals in plant disease management. Compared to synthetic chemical fungicides, the use of microbial antagonists or BCAs has become a “hot” topic due to the numerous advantages as non-hazardous, green, economical and feasible applications to control post-harvest pathogen infections (Bonaterra et al., 2012).

## Fungi as BCAs

Fungal BCAs are able to antagonize plant pathogens and protect their host plants. For example, several strains of *Trichoderma* have been developed as BCAs against the fungal pathogens *Penicillium*, *Fusarium, Aspergillus*, *Alternaria*, *Pythium*, *Rhizoctonia*, *Phytophthora*, *Pyricularia*, *Botrytis*, and *Gaeumannomyces* (Pal and Gardener, 2006; Adebola and Amadi, 2010; Agarwal et al., 2011; Alam et al., 2011; Nally et al., 2012). As a BCA, *Trichoderma* can suppress various air- and soil-borne plant pathogens; thus, can be conceivably used as biopesticides in greenhouse and/or field trials. According to Silva et al. (2017), certain strains of nematophagous fungi can manage the populations of *Meloidogyne enterolobii* in an integrated pest management (IPM) approach. AMF could also protect crops against soil-borne pathogens, including RKN, albeit the unclear mechanisms of antagonism (Vos et al., 2012).

The use of nematophagous and endoparasitic fungi has been deployed as antagonists to suppress RKN (Pendse et al., 2013). The talc-based formulation of the fungal BCA, *Paecilomyces lilacinus*, was found to be more active in reducing the population of *Meloidogyne incognita* in soils cultivated with tomato plants (Priya and Kumar, 2006). The efficiency of *P. lilacinus* in controlling nematodes was observed in several horticultural crops, including tomato, okra, and capsicum (Rao, 2007). The most widely used BCA for plant-parasitic nematodes is the fungus *P. lilacinus*, which has shown an appropriate replacement to synthetic chemical control in pre- and post-planting applications (Atkins et al., 2005). *P. lilacinus* infects eggs, juveniles and females of *M. javanica* by direct hyphal penetration (Esfahani and Pour, 2006). *P. lilacinus* can boost tomato yield while reducing the population of *M. incognita* in the soil and on the roots (Kalele et al., 2010). RKN management can also be achieved by using *P. lilacinus* and *Bacillus firmus* either individually or in combination. However, the mixture of *P. lilacinus* and *B. firmus* applied in soils 2 weeks prior to tomato transplantation showed the best practice to control *Meloidogyne* spp. (Anastasiadis et al., 2008).

Coating the seed with *Trichoderma viride* and *P. lilacinus* effectively reduced the nematode population in the soil. Species of *Aspergillus* and *Paecilomyces* were found to be antagonistic to *M. incognita* when compared to the single bio-agent treatment; thus, resulting in enhanced plant growth (Table 1; Bontempo et al., 2017). Kerry and Hidalgo-Diaz (2004) developed a management technique using the nematophagous fungus *Pochonia chlamydosporia* to manage RKN for the purpose of organic vegetable production. Okra seeds treated with *Trichoderma harzianum*, *T. viride*, *P. lilacinus*, *P. chlamydosporia*, and *P. fluorescens* at 20 g kg^−1^ seed signifcantly reduced the nematode population in the soil and promoted plant growth development (Kumar et al., 2012). Sharf et al. (2014) have reported that *P. chlamydosporia* exhibited nematicidal effects against *M. incognita* on infected common bean under greenhouse condition. *Trichoderma* spp. synthesizing chitinases, lytic enzymes, proteases, and glucanases were found to manage vegetable crop diseases (Punja and Utkhede, 2003). *T. harzianum*, *T. viride*, and *T. hamatum* have nematocidal properties when they colonize the roots of host plant and enhance their growth performance (Girlanda et al., 2001; Siddiqui and Shaukat, 2004; Zhang and Zhang, 2009). Because crop yield is mainly influenced by climatic conditions, agronomic factors, pests, and nutrient availability in the soil (Harman et al., 2004; Elrys et al., 2019a, 2020a), researchers must consider these factors in the selection of fungal BCAs.

Likewise, *Trichoderma* spp. can prevent nematode penetration and development in plants through the regulation of metabolites (Bokhari, 2009). Usman and Siddiqui (2012) have shown that the *M. incognita* and other RKN are more affected by the culture filtrate of *Trichoderma*. Species of *Trichoderma* can produce viridin, a nematicidal chemical (Watanabe et al., 2004). Gliotoxin and acetic acid, have also been reported as nematicidal substances in the culture filtrates of *T. virens* and *T. longibrachiatum,* respectively (Anitha and Murugesan, 2005). In response to *M. incognita*, *T. polysporum* has the ability to synthesize cyclosporine, the peptide that has a nematicidal action (Li et al., 2007). The efficacy of *P. lilacinus*, as bioagents or bioproducts in mixtures, significantly decreased the number of *M. incognita* on eggplant (Hanawi, 2016). It has been shown that different sources of N and carbon affect the growth and antagonism of *Trichothecium roseum* and *T. viride* (Arya, 2011). Although fructose and lysine were mostly effective against *T. viride*, rhamnose and glycine were more effective against *T. roseum*. There is an adverse effect of fungal culture filtrates on the egg hatching and juvenile mortality of RKN. Plants treated with the fungal BCA, *Lecanicillium muscarium*, decreased the number of galls in plants, eggs, juveniles (J2) and the reproduction factor (Rf) of *Meloidogyne hapla* compared to control plants (Hussain et al., 2017). In addition, plant growth was greatly improved when treated with *L. muscarium*. *Trichoderma* and *P. lilacinum* isolates dramatically reduced nematode egg number and mass, minimized root gal injury, and improved plant root mass development when compared to control plants without the fungal BCAs (Kiriga et al., 2018). Overall, more than 30 genera and 80 species of fungi can parasite RKN (Gaziea-Soliman et al., 2017). Prince et al. (2011) have demonstrated that the fungus, *Colletotrichum falcatum*, has antagonistic potential against the fungal pathogens *Penicillium citrinum*, *Botrytis cinerea*, and *Trichodermum glaucum*. Moreover, other fungi, such as *Ampelomyces speciosus* and *Acremonium alternatum*, have the ability to degrade the mycelia of fungal pathogens, indicating that not only rhizobacteria, but also fungi can serve as BCAs (Kiss, 2003).

## Mechanisms used by MBCAs

**Figure 2:**
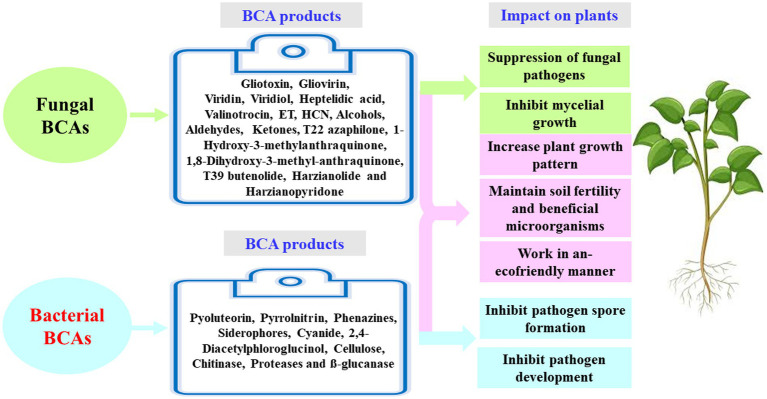
Fungal and bacterial biocontrol agents (BCAs) and their impact on plant. ET, ethylene; HCN, hydrogen cyanide.

**Figure 3:**
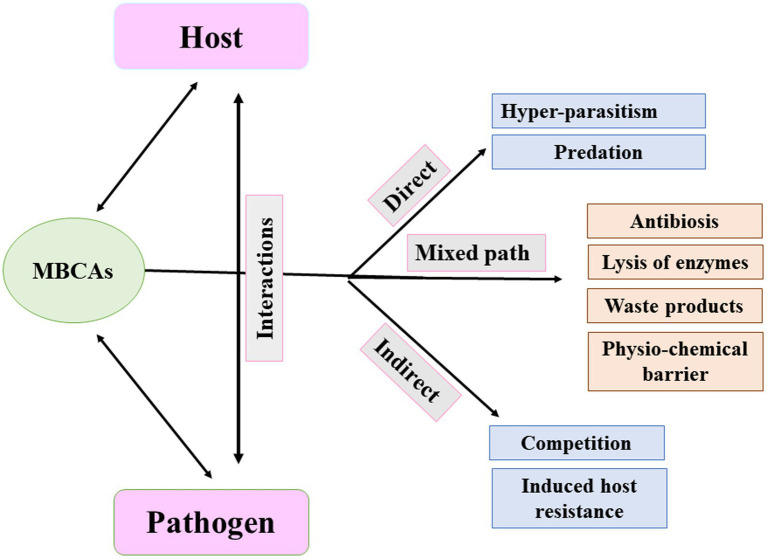
Pathways of microbial biocontrol agents (MBCAs).

Understanding the appropriate conditions for implementing proper programs against plant pathogens requires collaborations between different research groups focusing on the mechanisms associated with MBCAs to manage diseases on plants. In the last two decades, extensive research has focused on the antifungal effect, rhizosphere colonization, and crop benefits linked to MBCA (Compant et al., 2010; Al Hamad et al., 2021; Al Raish et al., 2021; Alwahshi et al., 2022). Thus, the products of MBCA on plant fungal pathogens and their impacts on plants are illustrated in Figure 2. The primary strategy of MBCA are summarized as antibiosis, competition for micronutrients such as iron, mycoparasitism, production of hydrolytic enzymes, and induction of ISR in host plants (Figure 3). In addition, the production of metabolites that are inhibitory to plant pathogenic rhizosphere microorganisms is considered one of the major biocontrol activities in many MBCA (Haas and Keel, 2003).

In several microorganisms, antibiosis, also known as secondary metabolites, results in the production of various toxic chemicals to pathogenic microorganisms; thus, they are suitable for the plant growth and development. An antibiotic-producing microorganism must manufacture the antibiotic in the correct microniche on the root surface to effectively control plant diseases (Lugtenberg and Kamilova, 2009). Actinobacteria (8,700 distinct antibiotics), bacteria (2,900), and fungi (4,900) can produce massive amounts of antibiotics (Bérdy, 2005). Mutagenesis has been reported to be successful in determining the role of antibiotics generated by bacterial BCA isolates to control pathogens associated with plant infections (Liu et al., 2007).

Ongena and Jacques (2008) have investigated the lipopeptides (surfactin, iturin, and fengycin) in *Bacillus* spp. It has also been reported that pyrrolnitrin, 2,4-diacetylphloroglucinol (DAPG), and phenazine can be potential antibiotic metabolites in *Pseudomonas* (Raaijmakers and Mazzola, 2012). *Pseudomonas* spp. have the ability to generate pyoluteorin, siderophores, and cyanide, among other antimicrobial chemicals (Compant et al., 2010). In addition, the enzymatic activity of cellulase, proteases, β-glucanase, and chitinase can lyse fungal cells (Hernandez-Leon et al., 2015). Antibiotic metabolites produced by *Pseudomonas* spp. are regulated by complex regulatory networks and high number of transcription factors (Berry et al., 2014). Significant classes of antifungal antibiotics are lipopeptides or peptides that are produced by the ribosomes or non-ribosomes of *Bacillus* spp. (Figure 2). Arseneault and Filion (2017) have discussed that antibiotics can be generated by BCA strains in soil.

*Bacillus* spp. have the ability to produce various biologically active chemicals that hinder the development of several crop diseases (Zhao et al., 2013). An investigation by Chowdhury et al. (2015) revealed that the quantity of antibacterial or antifungal chemicals produced by *Bacillus* spp. in the rhizosphere is somewhat little, causing doubts on the role of rapid management of plant diseases. Several isolates of *P. fluorescens* were found to generate cyclic lipopeptides (CLPs), such as viscosinamide, amphisin and tensin, that were effective against fungal pathogens, *R. solani* and *Pythium ultimum* (Nielsen et al., 2002).

**Figure 4:**
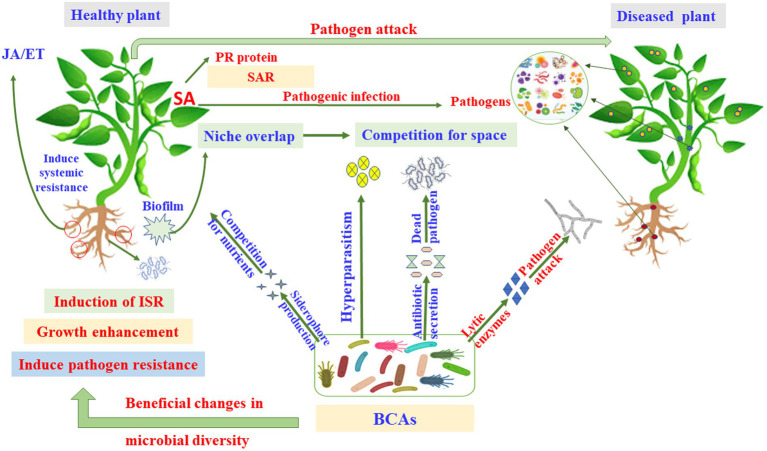
Pathways and modes of action of microbial biocontrol agents (MBCAs). ET, ethylene; ISR, induced systemic resistance; JA, jasmonic acid; PR, pathogenesis-related; SA, salicylic acid; SAR, systemic acquired resistance.

**Figure 5:**
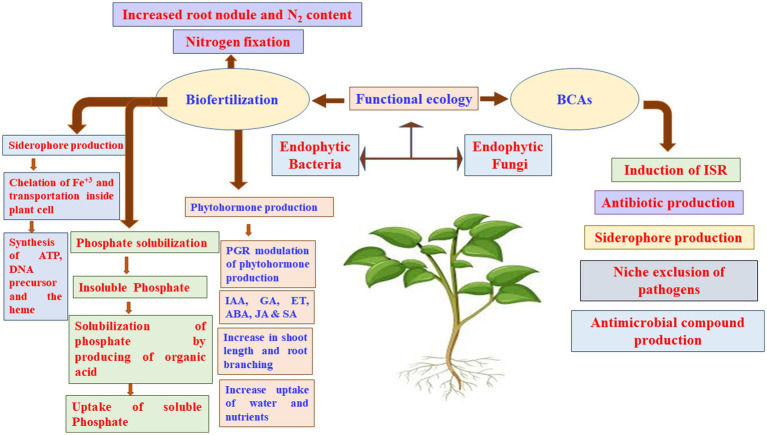
Dynamic microbial ecologies. ABA, abscisic acid; BCA, biocontrol agents; ET, ethylene; GA, gibberellic acid; IAA, indole acetic acid; ISR, induced systemic resistance; JA, jasmonic acid; PGR, plant growth regulators; SA, salicylic acid.

Biological control is an application of beneficial organisms, genes, and their products in the form of metabolites (Glare et al., 2012). Several *in vitro* metabolites of microorganisms were utilized to control pathogenic infections (Köhl et al., 2019). As a result, these secondary metabolites can be utilized as products of a BCA; and thus, they are effective to ameliorate the negative impact of other pathogenic microorganisms while also being environmentally friendly. Antimicrobial activities of some fungal BCAs may also exhibit antagonistic effects against fungi. For example, *Trichoderma* spp. are commonly found in soil and provide a variety of volatile and nonvolatile compounds. Volatile compounds, such as cyanide, hydrogen, ET, aldehydes, ketones and alcohols; and nonvolatile substances, such as peptides, can inhibit the mycelial growth in some pathogenic fungi. Many antifungal compounds, such as gliovirin, gliotoxin, viridiol, heptelidic acid, valinotrocin, and viridin can be produced by *Gliocladium virens*, which acts as a MBCA. Singh et al. (2005) demonstrated that gliotoxin can effectively reduce the fungal pathogens, *Pythium aphanidermatum*, *M*. *phaseolina*, *Pythium debaryanum*, *R. solani*, *Sclerotium rolfsii*, and *Rhizoctonia bataticola*. Vinale et al. (2009) have stated that the production of 1-hydroxy-3-methylanthraquinone, 1,8-dihydroxy-3-methyl-anthraquinone, T22 azaphilone, harzianolide, T39butenolide, and harzianopyridone by *T. harzianum* strains T22 and T39 has the ability to control the plant fungal pathogens *Leptosphaeria maculans*, *Phytophthora cinnamomi*, *R. solani*, *Botrytis cinerea*, and *P. ultimum*. Several secondary metabolites have been isolated and recognized by different methods such as high-performance liquid chromatography (HPLC) and gas chromatography–mass spectrometry (GC–MS). Shanthiyaa et al. (2013) have investigated three isolates, Cg-5, Cg-6, and Cg-7, that produce the secondary metabolite, chaetoglobosin A, in the culture filtrate detected by the UV spectrum at 250 nm. The antimicrobial compounds released by fungi may also control phytopathogens during post-harvest infections. The post-harvest infection causes excessive damages in vegetables and fruit (Figures 4, 5).

## Competition in the rhizosphere

Co-existence of two living microorganisms occurs when a population of a particular microorganism strives to achieve something greater, such as space or food supply (Stirling, 2017). Pathogenic and non-pathogenic microorganisms compete for food and resources in the rhizosphere. It has been known for a long time that non-pathogenic plant-associated bacteria are usually protected by colonizing plants and, as a result, this debilitate the limited available substrates and prevent the spread of the pathogens. The abilities of any microorganism to compete with others for essential nutrients and exudates secreted by the plant roots and their capability of colonizing into the root surface of host plants are termed rhizosphere competence. In the rhizosphere, the beneficial interactions between plants and microorganisms can regularly occur; thus, promoting growth and/or enhancing tolerance to biotic and abiotic stresses in plants (Zamioudis and Pieterse, 2012).

Rhizosphere competence can successfully establish microbial communities on or near the plant roots. Plant root colonization by PGPM can protect plants against pathogens and promote plant growth, and chemotaxis to root exudates is considered as an essential prerequisite for efficient root colonization (de Weert and Bloemberg, 2006). Microbial community in the rhizosphere is found to be important shortly after planting, but regularly decreases during the cropping season (Weller, 2007). Adesina et al. (2009) have reported *in vitro* antagonism of 15 *Pseudomonas* strains to *R. solani* in the rhizosphere. Only *Pseudomonas jessenii* RU47 has been effective to bottom rot disease on lettuce caused by *R. solani*. Tryptophan can stimulate the growth of adventitious roots and root hairs of the IAA-producing *B. subtilis* SRB28, which can colonize root tissues in sorghum, produce microcolonies, and persist in the rhizosphere (Das et al., 2010).

Rhizosphere microorganisms promoting plant growth, represent a wide range of species. PGPM are categorized according to their ability to colonize roots, survive, increase their numbers in the microhabitats on the root surface, compete with indigenous microorganisms, and increase resistance in host crops (Gamalero et al., 2004). PGPM can not only promote plant development, but also they are often used as BCAs to suppress plant diseases. The plant-associated *Bacillus*, *Pseudomonas*, *Lactobacillus* and actinobacteria strains are used as biofertilizers and BCAs in agriculture (Borriss, 2011; Sivasakthi et al., 2014; Lamont et al., 2017; Shivlata and Satyanarayana, 2017). Furthermore, *Acetobacter*, *Serratia*, *Azospirillum*, *Paenibacillus Burkholderia*, *Herbaspirillum*, and *Rhodococcus* can also enhance growth in crop plants (Babalola, 2010). Chakraborty et al. (2013) reported that a number of PGPR traits, such as production of siderophore, solubilization of phosphate, synthesis of IAA, and antagonism against fungal pathogens, were found to stimulate growth in tea plants. This has been linked with an increase in the number of shoots and leaves under greenhouse and field conditions. In general, soils with active microbial ecosystems and high organic matter require less fertilizer than soils without any microorganisms (Bender et al., 2016).

Biofertilizers made from microorganisms that help plants obtain their nutrients can colonize plant roots, to solubilize P, produce siderophore and HCN, and fix N_2_ (Figure 5; Pii et al., 2015; El-Sobky et al., 2022). N_2_ fixation by PGPR provides a considerable amount of N to the farming systems worldwide, with estimations ranging from 20 to 22 Tg N annually (Herridge et al., 2008), which may reach in some years to up to 40 Tg N (Galloway et al., 2008). Moreover, it has been reported that the biological N_2_ fixation may provide the African countries approximately 12 Tg N year^−1^ (Elrys et al., 2019b, 2020b). Crop yields might be limited by other nutritional elements, such as Fe and Zn. Similar to P, Fe is highly abundant in soils; yet, it is not available to plants in most cases. The synthesis of organic acids or siderophores by various PGPR strains increases Fe accessibility (Ahmed and Holmstrom, 2014).

Auxins are produced by a variety of PGPR (Gupta et al., 2015) that is involved in plant growth and development (Jha and Saraf, 2015) and plant architecture (Vacheron et al., 2013). The auxin, IAA, produced by PGPR has received much of attention. It is highly involved during PGPR-plant interactions (Afzal et al., 2015). Auxin-producing PGPR have been reported to cause transcriptional alterations in the hormone levels, resistance/tolerance to biotic/abiotic stress, and regulation of cell wall-linked genes (Spaepen et al., 2014). IAA may also increase root length (Hong et al., 1991), enhance root biomass, while reducing the size and density of stomata (Llorente et al., 2016). Plant growth and development can also be stimulated by the induction of auxin-response genes (Ruzzi and Aroca, 2015).

In addition, PGPRs can produce GA and CKs (Gupta et al., 2015), although the exact process remains unknown (Kang et al., 2009). A limited number of PGPR strains can produce huge amounts of GA; thus, significantly increase the shoot growth in plants (Jha and Saraf, 2015). Exudates are expected to contain organic acids, sugars, and amino acids, which are highly abundant in the cytoplasm of plants, but low quantities of complex secondary metabolites, including flavonoids, terpenes, and phenolic substances, which may attract certain rhizosphere microorganisms (Musilova et al., 2016). Plant health and physiology could be improved due to PGPR colonization of roots, resulting in more seeds and blooms (Kumar et al., 2016). According to Nivedhitha et al. (2008), actinobacteria isolated from the rhizosphere of bamboo was found to be capable to suppress the fungal pathogen, *Fusarium* sp., while boosting plant growth and development. Harzianic acid produced by *T. harzianum* not only promoted plant growth, but also showed antifungal effects against *Pythium irregulare*, *Sclerotinia sclerotiorum*, and *R. solani*, even at very low doses (Vinale et al., 2009). MBCA are important to the advancement and improvement of plant growth development, as well as the prevention of the attack of plant pathogens.

## Future perspectives

Biological control management is one of the most promising applications for sustainable agriculture. It is a proven to be eco-friendly agricultural pest control approach. This strategy uses living microorganisms to reduce the pest populations in a conservative, dependable, and ecologically amicable manner. In the developed countries, biological control is a remarkable tool to achieve sustainable, less expensive, and safe pest control management; thus, offering benefits to breeders and consumers when compared to synthetic (chemical) pest management. This review has provided an overview of antagonistic modes of action of MBCA, which are regarded as practical substitutions to synthetic fungicides as well as stimulation of plant growth and development for post-harvest purposes. Researchers working in the field of MBCA must anticipate new and distinct questions, in order to provide solutions that help in the development of novel biocontrol technologies/applications. Bioinformatics, molecular biology, analytical chemistry, and biostatistics have also shed lights on new research areas aimed at defining the MBCA-pathogen-plant interaction (Spadaro and Gullino, 2005).

One should not neglect the environmental conditions that also play a crucial role in the process of antagonism and the mode(s) of action of MBCA. The following conditions should be taken into consideration, when researchers isolate, identify and characterize a MBCA strain:

1. The spread of the infection associated with nematodes, fungi, and bacteria, as well as the potential antagonists in the micro- and macro-environment of the interaction.

2. The best conditions for the application of BCA.

3. The reaction of MBCA to the local communities and to various management strategies.

4. The limiting factors of effective colonization and articulation of biological control characteristics.

5. The plant components and dynamics that induce host defense.

## Conclusion

Many crops are affected by various pathogens. PGPM of pests and diseases in crops are generally regarded as a sustainable alternative for conventional chemical plant protection. These PGPR and PGPF acting as MBCAs are a safe, effective, and environmentally friendly form of pest management that do not harm the environment or the human health. PGPR/PGPF are antagonistic microorganisms that could be exploited as biopesticides and biofertilizers for better plant health and growth improvement. Adoption of PGPR/PGPF-based biopesticides/biofertilizers on a commercial scale may substantially contribute to sustainable agriculture and safe environment. This review has provided an overview on the research related to PGPMs, their benefits and effects as potential bioinoculants for plant growth and biological control. The increased use of PGPMs requires the achievement of accurate selection of beneficial PGPR/PGPF strains and consortia, the mechanisms underlying PGPM-plant interactions, and the ability to prepare for future agricultural challenges.

## Author contributions

All authors listed have made a substantial, direct, and intellectual contribution to the work and approved it for publication.

## Funding

This project was funded by Abu Dhabi Education and Knowledge (Grant #: 21S105) to KE-T and Khalifa Center for Genetic Engineering and Biotechnology-UAEU (Grant #: 31R286) to SA.

## Conflict of interest

The authors declare that the research was conducted in the absence of any commercial or financial relationships that could be construed as a potential conflict of interest.

## Publisher’s note

All claims expressed in this article are solely those of the authors and do not necessarily represent those of their affiliated organizations, or those of the publisher, the editors and the reviewers. Any product that may be evaluated in this article, or claim that may be made by its manufacturer, is not guaranteed or endorsed by the publisher.
